# A Heart Segmentation Algorithm Based on Dynamic Ultrasound

**DOI:** 10.1155/2022/1485584

**Published:** 2022-06-17

**Authors:** Mingjun Tian, Minjuan Zheng

**Affiliations:** Department of Ultrasound, Xijing Hospital, Fourth Military Medical University, Xi'an 710032, China

## Abstract

The heart is one of the most important organs of the human body. The role of the heart is to promote blood flow and provide sufficient blood flow to organs and tissues. The research on the heart has important theoretical and clinical significance. Because of the noninvasive and intuitive display of ultrasound image, it can dynamically obtain the heart state and has become the main means to detect the heart dynamics. We analyze the characteristics of cardiac ultrasound image from the medical point of view and signal processing. The heart movement is periodic and rhythmic. The image signal can be decomposed. Firstly, the image is decomposed into high- and low-frequency signals to highlight different dimensional information. Then, the attention model was introduced, focusing on the heart region. Finally, the multidimensional network carrying model was established to achieve cardiac segmentation. The experimental results show that the AOM of the algorithm proposed in this paper reaches 92%, which has a certain degree of advancement and can assist doctors to make accurate diagnosis.

## 1. Introduction

The heart is an important organ of the human body. Its main function is to provide pressure for blood flow and run the blood to all parts of the body. Its efficiency is related to the shape and size of the heart [1]. Therefore, it is very important to extract the region of the heart and carry out research. Tang et al. [2] proposed a clustering algorithm for heart sound segmentation. Icardo [3] established a model from morphology to analyze the heart. Ahn et al. [4] focused on the region of coronary artery through CT and carried out research. Mythili et al. [5] used SVM to predict heart disease. Pedrosa et al. [6] built a model to analyze the voice of young children. Methaila et al. [7] used the big data technology to analyze early heart disease. Pace et al. [8] implemented the whole heart segmentation interactively. Saquib et al. [9] diagnosed heart disease by calculating the volume ratio. Xiong et al. [10] combined CT images with clinical data for analysis. Wolterink et al. [11] used dilated convolutional neural networks to segment MR images. Arabasadi et al. [12] used a hybrid neural network genetic algorithm to predict heart disease. Tong et al. [13] constructed 3D deep supervised U-Net to segment all hearts. Ahmed et al. [14] built a deep network to segment the heart. Gao and Lu [15] focused on fetal baseline to realize classification and extraction. Xu et al. [16] combined a deep learning network and graph matching to realize whole heart segmentation. de Albuquerque et al. [17] proposed fast heart fat segmentation based on CT data set. Naseer et al. [18] constructed fuzzy sets to diagnose heart diseases. Yoshida et al. [19] used U-Net to automatically segment the heart based on CT data. Banerjee et al. [20] reconstructed the heart in 3D from 2D data. Diniz et al. [21] built Concat-U-Net to realize automatic heart extraction. Diniz et al. [22] built U-Net++ to realize heart segmentation. Liu et al. [23] proposed automatic segmentation algorithm using attentional convolutional network. Chen et al. [24] constructed 3D filter to suppress ultrasonic image noise. Song et al. [25] proposed deep networks for heart segmentation and explained the significance and challenges of heart segmentation.

Through the above introduction, the current analysis of the heart is focused on mature images with high imaging quality such as CT [26] and MR [27]. However, CT and MR mostly present static images, which cannot meet the situation of dynamic analysis. Therefore, medical staff often use dynamic echocardiography in analyzing [28] and has achieved good results. Echocardiography is the most widely used cardiac examination method in clinic, which can dynamically evaluate the structure and function of the heart. The ventricular wall motion of the heart is an important driving force to maintain the function of the heart pump. It is very important for doctors to identify whether the ventricular wall motion is normal based on ultrasonic images. The current ventricular wall motion evaluation is mainly by eye or manual trace for motion amplitude, which is very dependent on the operator's experience and time-consuming. If the segmental recognition method of ventricular wall can be established to quickly identify the abnormal motion of myocardium, it would be very helpful for the diagnosis of heart disease.

However, due to the large noise interference of ultrasonic image and the lower imaging quality than CT and MR, it is difficult to put forward computer-aided segmentation. In general, main problems are as follows: (1) low image quality leads to limited access to information. (2) The heart only occupies a limited area in the image, which is inefficient to process the image pixels in a unified scale. (3) The constructed cardiac ultrasound image feature network has limited carrying capacity and insufficient representation.

In view of the above shortcomings, we propose a new segmentation algorithm based on dynamic echocardiography: (1) Octconv is proposed from the perspective of signal composition, and the signal is decomposed into high- and low-frequency parts to extract different frequency information; (2) to simulate the doctor's diagnosis process and build an attention mechanism to focus on the region of the heart; and (3) from the perspective of deep learning, a multidimensional information carrying network is constructed to fuse low-frequency and high-frequency signals to achieve heart segmentation.

## 2. Algorithm

**Figure 1:**
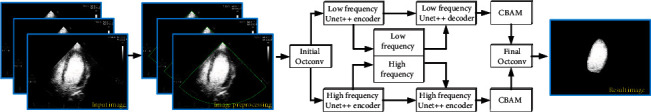
Algorithm flowchart.

The research of echocardiography algorithm is based on the current situation of low echocardiography quality and the actual situation of echocardiography clinical diagnosis. A dynamic echocardiography segmentation algorithm based on deep learning is constructed, and the specific block diagram is shown in Figure 1. Firstly, Octconv is proposed to decompose the image into low-frequency and high-frequency signals by simulating the principle of visual perception. Then, an attention mechanism is built to focus on the region where the heart is located. Finally, a multidimensional information carrying network is built to realize heart segmentation.

### 2.1. Image Preprocessing

Evaluating the motion of ventricular wall is an important content of echocardiography. The left ventricle is the main chamber responsible for pumping blood. Usually, the left ventricular wall is divided into 16 or 17 segments (including the apex) according American Society of Echocardiography (ASE) guidelines. Each segment was dominated by its corresponding coronary artery branches for blood supply. Segmental wall motion abnormalities can occur in coronary heart disease or other myocardial diseases (diabetes, hypertension, amyloidosis, etc.); thus, each LV segment is needed to be evaluated. This cardiac segmentation can help doctors quickly identify the region of ventricular wall with abnormal motion, so as to accurately locate the location of impaired myocardium.

**Figure 2:**
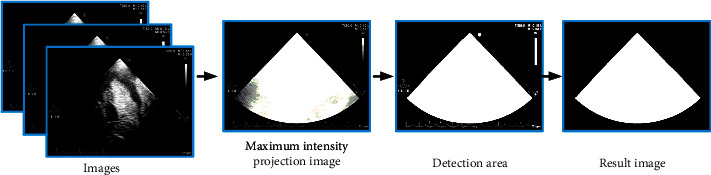
Image preprocessing flowchart.

We used video sequences to do our research. Therefore, the detection region is extracted from the pixel variation of the whole video sequence, and the sector region can be preliminarily determined. To this end, we build the maximum density projection mechanism to obtain the information of the sequence image and focus on the image region, as shown in Figure 2.

The projection of maximum density is the maximum value of the coordinates at (*x*, *y*) and can be used to calculate the area of concern.

(1) $$\begin{array}{c} M\left(x,y\right)=\max\left(I_{1}\left(x,y\right),.\cdots I_{n}\left(x,y\right)\right). \end{array}$$

After the maximum density projection of the image, the threshold method was used to determine detection area, and then, the ultrasonic image region was obtained according to mathematical morphology operation.

### 2.2. Octconv

**Figure 3:**
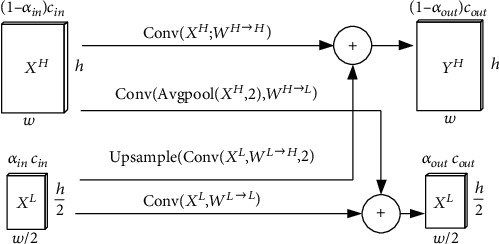
Octconv composition.

Information can be transmitted at different frequencies, and the characteristic diagram output by the convolution layer can also be regarded as the combination of information at different frequencies. Therefore, scholars have proposed Octconv to store and process feature graphs with low spatial resolution and slow spatial change. Octconv has orthogonality and complementarity and can establish a better topology to reduce the spatial redundancy caused by low-frequency information in deep convolution network. The specific structure is shown in Figure 3. *α*_in_ is the proportion of input low-frequency channel in the total channels. The corresponding input size is *c*_in_ × *w* × *h*, and the output size is *c*_out_ × *w* × *h*. *W* is the convolution kernel, and the corresponding parameter is *c*_in_ × *c*_out_ × *k* × *k*, stride = 1, and padding = same. The most available calculation amount is (*c*_in_ × *k* × *k*) × (*c*_out_ × *w* × *h*). Compared with ordinary convolution, Octconv contains a certain proportion of low-frequency channels, and the complexity is improved. But the performance is better than ordinary convolution.

Octconv uses the coefficient *α* to append the feature graph into high-resolution component (*X*^H^) and low-resolution component (*X*^L^). *X*^H^ saves the edge, contour, and other details of the images with a large amount of information data. *X*^L^ saves the abstract information of the images with a small amount of information data. The multifrequency feature representation method is constructed, and the smooth low-frequency mapping is stored in the low-frequency tensor to reduce spatial redundancy. The specified convolution kernel *w* is used to convolute *x* to obtain the corresponding component:

(2) $$\begin{array}{c} Y^{H⟶H}=\text{Conv}\left(X^{H},W^{H⟶H}\right),\\ Y^{H⟶L}=\text{Conv}\left(\text{Avgpool}\left(X^{H},2\right),W^{H⟶L}\right),\\ Y^{L⟶H}=\text{upsample}\left(\text{Conv}\left(X^{L},W^{L⟶H}\right),2\right),\\ Y^{L⟶L}=\text{Conv}\left(X^{L},W^{L⟶L}\right). \end{array}$$

High-frequency output is *Y*^*H*^ = *Y*^*H*⟶*H*^ + *Y*^*L*⟶*H*^, and low-frequency output is *Y*^*L*^ = *Y*^*H*⟶*L*^ + *Y*^*L*⟶*L*^. The amount of Octconv convolution calculation is only 1/4 of that of ordinary conv.

### 2.3. CBAM Attention Module

**Figure 4:**
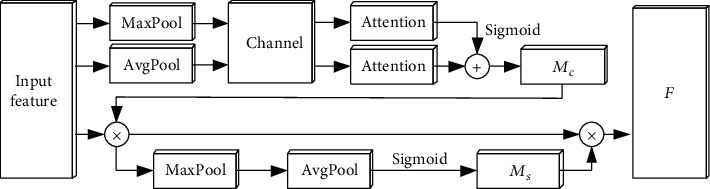
CBAM structure.

Due to the increase of the length of neural network, the long-distance information will be weakened, resulting in the loss of important information after information transmission. To reduce the risk of losing important information, we focus our limited attention on important information to build attention modules. Due to the convolutional block attention module (CBAM) combining channel and space, the channel attention module and spatial attention module are connected in series. According to the importance of features, they focus on the area of interest step by step and extract key information through corresponding spatial transformation. The structure is shown in Figure 4.

1. Channel attention module. Input feature *F* (*H* × *W* × *C*) and two channel features (1 × 1 × *C*) are obtained through Maxpool and Avgpool to connect MLP (multilayer perceptron). The corresponding number of neurons in the first layer is *C*/*r*, and the number of neurons in the second layer is *c*. The weight coefficient of the activation function is obtained as follows:

(3) $$\begin{array}{c} M_{c}\left(F\right)=\text{Sigmoid}\left(\text{MLP}\left(\text{AvgPool}\left(F\right)\right)+\text{MLP}\left(\text{MaxPool}\left(F\right)\right)\right) \end{array}$$

The corresponding new feature is

(4) $$\begin{array}{c} F^{\prime }=M_{C}\left(F\right)F. \end{array}$$

(2) Spatial attention module. Maxpool and Avgpool are performed on *F*′ to get the channel description, and the dimension is *H* × *W* × 1. Splice the two descriptions according to the channel, and pass through 7 × 7 convolution, and the weight function is

(5) $$\begin{array}{c} M_{s}\left(F^{\prime }\right)=\text{Sigmoid}\left\{f\left(\text{AvgPool}\left(F^{\prime }\right),\text{MaxPool}\left(F^{\prime }\right)\right)\right\} \end{array}$$

The corresponding new feature is

(6) $$\begin{array}{c} F^{\prime \prime }=M_{s}\left(F^{\prime }\right)F^{\prime }, \end{array}$$

where *M*_*c*_ and *M*_*s*_ form a complementary relationship. The use of Maxpool and Avgpool increases more diversified information, resulting in less computation and stable performance improvement of CBAM.

### 2.4. Network Model

The main advantage of U-Net++ is that it connects each branch U-Net to share a coding layer and allows the middle part of the model to participate in training, so that the information loss in the encoder process can be repaired to some extent. The performance is improved on the basis of limited increase of parameters. Since each branch U-Net shares an encoder path, the same information is lost in the downsampling process. U-Net++ obtains U-Net features with different depths in the decoder process, but this feature difference is obtained after each branch U-Net performs its own upsampling, so the information supplement of U-Net++ in the feature recovery process is limited and has lack of pertinence.

Through the above analysis, there are five layers in total based on the U-Net++ framework. Octconv is used to replace the traditional Conv2D. The whole framework is divided into two U-Net++ structure branches of high-frequency and low-frequency synchronous parallel. Octconv is used in each layer of coding-decoding to exchange low-frequency and high-frequency information. It reduces the model parameters, reduces the information loss introduced by each coding layer in downsampling, and makes the whole network obtain more abundant information. In the decoding stage, bilinear interpolation sampling is used to realize image restoration. After each sampling, it is spliced with the features of the same layer and the same scale and then connected with CBAM to strengthen the attention of convolution operation in the model to the target area and realize more accurate pixel category classification. The problem of offset of restoration features is avoided as much as possible in order to make the network more robust.

Init-Octconv and input high frequency obtain characteristics through downsampling, and then, Octconv is performed to obtain high-frequency and low-frequency output. COM-Octconv is input by high-frequency and low-frequency characteristics, and Octconv is directly carried out through hyperparameter *α* to control the proportion of high- and low-frequency channels of output. Fin-Octconv is input by high-frequency and low-frequency characteristics. After convolution, the low-frequency upsampling is added to the high frequency to output the high frequency.

We introduce the objective function. Tversky coefficient is the generalized coefficient of Dice coefficient and Jaccard coefficient:

(7) $$\begin{array}{c} TI\left(X,Y\right)=\frac{X\cap Y}{X\cup Y+\beta \left|X-Y\right|+\alpha \left|X+Y\right|}, \end{array}$$

where *X* represents the true value and *Y* represents the predicted value. In medical image segmentation, Dice Loss (*α* = *β* = 0.5) is often used in small lesion segmentation as the objective function. It has good performance in the case of extremely unbalanced samples, but in general, its use will have an adverse impact on back propagation and make the training unstable. As a result, the effect of Dice Loss is not ideal and the fluctuation range is large.

Therefore, we construct a new objective function to reduce the weight of simple samples and increase the weight of difficult samples. *γ* coefficient is introduced to learn difficult samples with small sample regions of interest:

(8) $$\begin{array}{c} FTL=\underset{c}{\sum }{\left(1-{TI}_{c}\right)}^{\gamma },\gamma \in \left[1,3\right]. \end{array}$$

## 3. Experiment and Result Analysis

**Figure 5:**
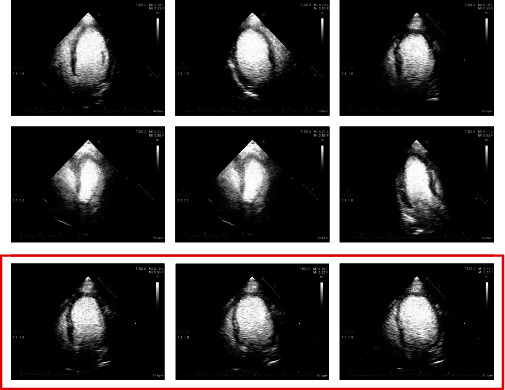
Data display.

The experiment is conducted with 50 groups of image sequences collected by the hospital, and the data resolution is 600 × 800, and the equipment is Philips Epiq 7c. As shown in Figure 5, the red box is the continuous image sequence. The area where the heart is located is marked at the pixel level by a professional doctor. We adopt double-blind method, which is a common algorithm in annotation field. The gold standard was determined by combining the labeling results of two physicians. The programming environment is Linux and Python. We adopted the deep learning network. So the input of the deep learning network we designed is 600 × 800. For other resolutions, normalize to 600 × 800. We built 1 : 1 ratio of training samples to test samples. The cross-validation method is adopted.

### 3.1. Octconv Performance

Based on the principle of signal processing, we decompose signals into low-frequency and high-frequency components to construct Octconv. To verify Octconv performance, we build a unified network model and compare the convergence curves of Octconv and ordinary convolution to obtain the performance. AOM is introduced to measure the convergence effect.

(9) $$\begin{array}{c} \text{AOM}=\frac{A\cap B}{A\cup B}. \end{array}$$

**Figure 6:**
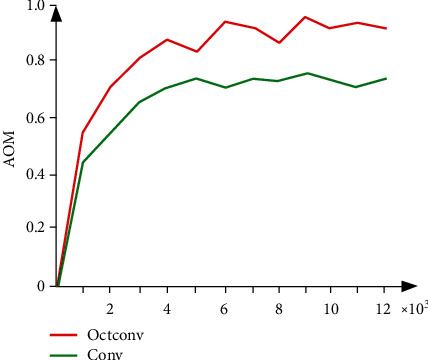
Algorithm convergence curve.

As shown in Figure 6, Octconv has better iteration times than traditional convolution when the curve converges. Due to the orthogonality and complementarity of Octconv, signals are decomposed into low-frequency and high-frequency components, which is in line with the principle of visual sensing and has low redundancy. However, the traditional convolution only considers the relationship between parts, resulting in a large amount of redundancy in the parameters and slow convergence speed.

### 3.2. Segmentation Effect Comparison

The loss function, crossentropy loss function, Dice Loss, and Tversky Loss are proposed as the objective function for experiments. We introduce AVM, AUM, and CM to measure the accuracy of the algorithm:

(10) $$\begin{array}{c} \left\{\begin{array}{c} \text{AVM}=\frac{A-B}{A},\\ \text{AUM}=\frac{B-A}{B},\\ \text{CM}=\frac{1}{3}\left\{\text{AOM}+\left(1-\text{AVM}\right)+\left(1-\text{AUM}\right)\right\}, \end{array}\right. \end{array}$$

where*A*is the segmentation result marked by the doctor and*B*is the segmentation result of the algorithm, in which AOM and CM are proportional to the segmentation result and AVM and AUM are inversely proportional to the segmentation result. The performance is shown in Table 1. Crossentropy loss is used in multitarget segmentation and analyzed from the perspective of energy, but the signal-to-noise ratio of ultrasonic image is low and the energy is not concentrated, resulting in poor performance. Dice Loss is often used in medical small target segmentation loss function, but the image area occupied by heart data is large, which has the risk of overfitting, resulting in inaccurate boundary focusing. Tversky comprehensively considers the differences between simple samples and complex samples and constructs weights, which has better improved the case of fuzzy boundary and reduced the probability of pixel misclassification.

**Figure 7:**
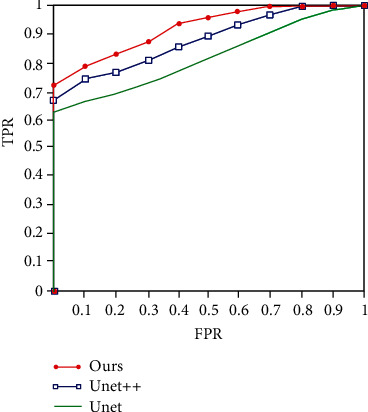
ROC curve.

In order to verify the performance of different network algorithms, Sen and Spe are introduced to measure:

(11) $$\begin{array}{c} \left\{\begin{array}{c} \text{Sen}=\frac{\text{TP}}{\text{TP}+\text{FN}},\\ \text{Spe}=\frac{\text{TP}}{\text{TP}+\text{FP}}, \end{array}\right. \end{array}$$

where TP represents the number of pixels correctly predicted as positive samples and TN represents the number of pixels correctly predicted as negative samples. FP represents the number of pixels incorrectly predicted as positive samples, and FN represents the number of pixels incorrectly predicted as negative samples. The results are shown in Table 2, and the corresponding ROC curve is shown in Figure 7.

**Figure 8:**
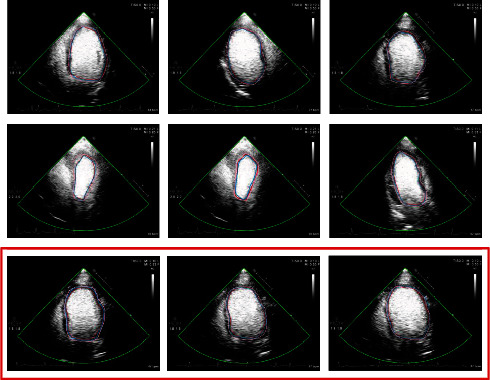
Segmentation effect.

To visually display the segmentation effect, as shown in Figure 8, the pretreatment results are shown in green, and the obtained sector area is the detection area.

The heart is periodic diastolic and contractile, which can be determined according to the correlation of motion. It is on this basis that the significance region is determined to achieve auxiliary segmentation. Traditional U-Net can inevitably lose details after downsampling. Encoder-decoder structure can reduce the loss, but after the image restoration by upper sampling, it is difficult to pay attention to the details and determine the contour limits of the target category under the influence of multinoise. The proposed attention model pays more attention to the target region, highlights the difference between foreground and background, and realizes accurate segmentation.

In this paper, the deep learning network is adopted, and the results are related to the input image. Therefore, if the acquisition equipment is changed, the image quality will be different and the algorithm should be retrained, but the overall framework remains unchanged, so the algorithm should have certain effects.

## 4. Conclusion

The heart is the human body's important organ and has very important sense to the dynamic monitoring. Based on dynamic echocardiographic noninvasive, can display the superiority, is currently the main observation way of the heart. However, it is limited by the mechanism of ultrasonic imaging; imaging noise is large and easy to produce motion blur. We propose a new heart segmentation algorithm, which decomposes the image into low-frequency and high-frequency signals according to the principle of signal decomposition to obtain different frequency information. According to the principle of cardiac dynamic contraction, the attention model was introduced to simulate the diagnosis process of doctors and focus on the region of the heart. The network model will be built to realize multiscale information carrying and finally realize heart segmentation. Subsequent studies on myocardial extraction and analysis will be carried out to study the lightweight network, improve the calculation efficiency of the algorithm, and quickly assist doctors to make accurate diagnosis.

## Figures and Tables

**Table 1:** Algorithm performance.

Algorithm	AOM	AVM	AUM	CM
Cross entropy	0.72	0.31	0.35	0.69
Dice	0.78	0.27	0.33	0.73
Tversky	0.92	0.18	0.21	0.84

**Table 2:** Algorithm performance.

Algorithm	Sen (%)	Spe (%)
U-Net	82	79
U-Net++	87	82
Ours	94	85

## Data Availability

The data used to support the findings of this study are available from the corresponding author upon request.

## References

[1] Des Jardin J. T., Chikwe J., Hahn R. T., Hung J. W., Delling F. N. (2022). Sex differences and similarities in valvular heart disease. *Circulation Research*.

[2] Tang H., Li T., Qiu T., Park Y. (2012). Segmentation of heart sounds based on dynamic clustering. *Biomedical Signal Processing and Control*.

[3] Icardo J. M. (2012). The teleost heart: a morphological approach. *Ontogeny and Phylogeny of the Vertebrate Heart*.

[4] Ahn S. J., Kang D. K., Sun J. S., Yoon M. H. (2013). Accuracy and predictive value of coronary computed tomography angiography for the detection of obstructive coronary heart disease in patients with an Agatston calcium score above 400. *Journal of Computer Assisted Tomography*.

[5] Mythili T., Mukherji D., Padalia N., Naidu A. (2013). A heart disease prediction model using SVM-decision trees-logistic regression (SDL). *International Journal of Computer Applications*.

[6] Pedrosa J., Castro A., Vinhoza T. T. Automatic heart sound segmentation and murmur detection in pediatric phonocardiograms.

[7] Methaila A., Kansal P., Arya H., Kumar P. (2014). Early heart disease prediction using data mining techniques. *Computer Science & Information Technology Journal*.

[8] Pace D. F., Dalca A. V., Geva T., Powell A. J., Moghari M. H., Golland P., Navab N., Hornegger J., Wells W., Frangi A. (2015). Interactive whole-heart segmentation in congenital heart disease. *International Conference on Medical Image Computing and Computer-Assisted Intervention*.

[9] Saquib N., Papon M. T. I., Ahmad I., Rahman A. Measurement of heart rate using photoplethysmography.

[10] Xiong G., Sun P., Zhou H. (2017). Comprehensive modeling and visualization of cardiac anatomy and physiology from CT imaging and computer simulations. *IEEE Transactions on Visualization and Computer Graphics*.

[11] Wolterink J. M., Leiner T., Viergever M. A., Išgum I. (2016). Dilated convolutional neural networks for cardiovascular MR segmentation in congenital heart disease. *Reconstruction, Segmentation, and Analysis of Medical Images*.

[12] Arabasadi Z., Alizadehsani R., Roshanzamir M., Moosaei H., Yarifard A. A. (2017). Computer aided decision making for heart disease detection using hybrid neural network-genetic algorithm. *Computer Methods and Programs in Biomedicine*.

[13] Tong Q., Ning M., Si W., Liao X., Qin J. (2018). 3D deeply-supervised U-Net based whole heart segmentation. *International Workshop on Statistical Atlases and Computational Models of the Heart*.

[14] Ahmed R. S., Liu J., Fei Z., Zahid M. (2018). Automated segmentation of whole cardiac CT images based on deep learning. *International Journal of Advanced Computer Science and Applications*.

[15] Gao W., Lu Y. Fetal heart baseline extraction and classification based on deep learning.

[16] Xu X., Wang T., Shi Y. Whole heart and great vessel segmentation in congenital heart disease using deep neural networks and graph matching.

[17] de Albuquerque V. H. C., Rodrigues D. D. A., Ivo R. F. (2020). Fast fully automatic heart fat segmentation in computed tomography datasets. *Computerized Medical Imaging and Graphics*.

[18] Naseer I., Khan B. S., Saqib S., Tahir S. N., Tariq S., Akhter M. S. (2020). Diagnosis heart disease using Mamdani fuzzy inference expert system. *EAI Endorsed Transactions on Scalable Information Systems*.

[19] Yoshida A., Lee Y., Yoshimura N., Kuramoto T., Hasegawa A., Kanazawa T. (2021). Automated heart segmentation using U-Net in pediatric cardiac CT. *Measurement: Sensors*.

[20] Banerjee A., Camps J., Zacur E. (2021). A completely automated pipeline for 3D reconstruction of human heart from 2D cine magnetic resonance slices. *Philosophical Transactions of the Royal Society A*.

[21] Diniz J. O. B., Ferreira J. L., Cortes O. A. C., Silva A. C., de Paiva A. C. (2022). An automatic approach for heart segmentation in CT scans through image processing techniques and Concat-U-Net. *Expert Systems with Applications*.

[22] Diniz J. O. B., Dias Júnior D. A., da Cruz L. B. (2022). Heart segmentation in planning CT using 2.5 D U-Net++ with attention gate. *Computer Methods in Biomechanics and Biomedical Engineering: Imaging & Visualization*.

[23] Liu H., Chu W., Wang H. (2020). Automatic segmentation algorithm of ultrasound heart image based on convolutional neural network and image saliency. *IEEE Access*.

[24] Chen H., Xu H., Shi P. (2021). 3-D Gabor-based anisotropic diffusion for speckle noise suppression in dynamic ultrasound images. *Physical and Engineering Sciences in Medicine*.

[25] Song Y., Ren S., Lu Y., Fu X., Wong K. K. (2022). Deep learning-based automatic segmentation of images in cardiac radiography: a promising challenge. *Computer Methods and Programs in Biomedicine*.

[26] Rahaman M. M., Li C., Yao Y. (2020). Identification of COVID-19 samples from chest X-ray images using deep learning: a comparison of transfer learning approaches. *Journal of X-Ray Science and Technology*.

[27] Oyama-Manabe N., Aikawa T., Tsuneta S., Manabe O. (2022). Clinical applications of 4D flow MR imaging in aortic valvular and congenital heart disease. *Magnetic Resonance in Medical Sciences*.

[28] Lisi M., Cameli M., Mandoli G. E. (2022). Detection of myocardial fibrosis by speckle-tracking echocardiography: from prediction to clinical applications. *Heart Failure Reviews*.

